# Are There Trigger Points in the Spastic Muscles? Electromyographical Evidence of Dry Needling Effects on Spastic Finger Flexors in Chronic Stroke

**DOI:** 10.3389/fneur.2020.00078

**Published:** 2020-02-21

**Authors:** Zhiyuan Lu, Amy Briley, Ping Zhou, Sheng Li

**Affiliations:** ^1^Department of Physical Medicine and Rehabilitation, McGovern Medical School, University of Texas Health Science Center at Houston, Houston, TX, United States; ^2^TIRR Memorial Hermann Hospital, Houston, TX, United States

**Keywords:** spasticity, stroke, trigger points, EMG, dry needling

## Abstract

The purpose was to examine the immediate effects of dry needling to spastic finger muscles in chronic stroke. Ten chronic stroke patients with spasticity in finger flexors participated in this experiment. Dry needling to the flexor digitorum superficialis (FDS) muscle was performed under ultrasound guidance for about 30 s (about 100 times). Clinical assessment and intramuscular needle EMG readings were made before and immediately after dry needling. Immediately after needling, the FDS muscle was felt less tight to palpation and the proximal phalangeal joint rested in a less flexed position (*p* = 0.036). The MAS score decreased for FDS (*p* = 0.017) and flexor digitorum profundus (FDP) (*p* = 0.029). Motor unit action potential (MUAP) spikes decreased from 41.6 ± 5.5 to 6.7 ± 2.2 spikes/s (*p* = 0.002), an 84% reduction after dry needling. However, the pre-needling spike frequency was not correlated to MAS or resting position of the FDS muscles. Dry needling to the spastic finger flexors leads to immediate spasticity reduction, increased active range of motion, and decreased frequency of motor unit spontaneous firing spikes. The results suggest that latent trigger points possibly exist in spastic muscles and they contribute partly to spastic hypertonia of finger flexors in chronic stroke.

## Introduction

Spasticity is a common disabling motor impairment after stroke. Though not fully understood, spasticity is a result of disinhibited descending excitatory inputs to spinal reflex circuitry, adaptive changes in intraspinal network and peripheral changes in spastic muscles (1). These excitatory inputs at least in part lead to hyperexcitable or spontaneous firing of motor units of spastic muscles (2). Adaptive changes occur in parallel, such as muscle fiber shortening and stiffening (3). They interact with each other in a vicious cycle, thus contributing to muscle overactivity and spasticity (4). Spasticity amplifies other motor impairments, e.g., weakness, and imposes significant limitations in patient's mobility and activities of daily living (5).

A wide spectrum of treatment options is available, including physical modality, stretching, oral medications to botulinum toxin injection and surgery to target different factors. Recently, there are clinical observational reports of dry needling for spasticity management after stroke. Dry needling is largely known to be effective for management of myofascial pain through breakdown of taut bands of trigger points (6). Unlike commonly used botulinum toxin therapy, dry needling causes immediate and short-term spasticity reduction, increased active range of motion, and improved gait (7). It remains unclear whether dry needling-induced spasticity reduction is mediated through the same mechanisms as for trigger points. The purpose of this study was to examine the immediate effects of dry needling to spastic finger muscles in chronic stroke and to explore the potential underlying mechanisms with intramuscular needle EMG recordings and analysis.

## Methods

### Study Design and Participants

Ten chronic stroke survivors with spasticity in finger flexors participated in this experiment (6 males and 4 females; average: 61.1 ± 4.1 years of age; 5 right spastic hemiplegia, and 5 left spastic hemiplegia). Time since stroke ranged from 6 months to 8 years, with an average of 4.6 ± 0.7 years. These patients were scheduled to receive botulinum toxin injections, including to their finger flexors. They gave written consent prior to dry needling. The data were retrospectively analyzed with approval from local ethic committee.

Dry needling to the FDS muscle was performed prior to botulinum toxin injections. A 27 gauge botulinum toxin injection needle connected to the EMG machine (Nicolet Viasys Viking system, sampling rate at 48 k Hz with a band-pass filtering from 20–10 K Hz) was used. This type of needle is specifically designed and manufactured for intramuscular recording, stimulating and injection. The needle was inserted into the FDS muscle (typically 1.2–2.5 cm in depth) from the taut band found by palpation. The needle tip position was visualized under ultrasound imaging (M Turbo, SonoSite, Bothell, WA), and was further verified by stretching of the fingers at the proximal interphalangeal (PIP) joints that induced increase in EMG amplitude and frequency. Continuous dry needling was performed by the injecting physician under ultrasound guidance for about 30 s (about 100 pokes).

Measurements of modified Ashworth scale (MAS), passive and active range of motion (PROM/AROM), and resting position of finger flexors were taken in the 3rd metacarpal phalangeal (MCP), PIP and distal interphalangeal (DIP) joints before and immediately (between 5 and 10 min) after dry needling. Resting position was measured using a goniometer when the subject's hand was rested on a table. MAS for FDS was estimated by ranging the PIP joint, i.e., stretching the FDS muscle individually, while stabilizing the subject's wrist and MCP joints at the naturally resting positions. MAS for FDP was estimated by ranging the DIP joint, i.e., stretching FDP specifically, while stabilizing the shaft of the middle phalange. Firmness of taut band of FDS muscles was assessed as well. These clinical assessments were performed by an independent, experienced physical therapist. The body configuration, the wrist joint position in particular, was maintained the same before and after needling. Intramuscular needle EMG readings from FDS were taken before and immediately after dry needling to measure spikes of spontaneous motor unit action potentials (MUAPs). In order to reduce the shift of recording area before and after dry needling, the needle tip location was visually seen under ultrasound guidance, and the depth and location was verified by the markers on the screen.

### Electromyogram Analysis

**Figure 1:**
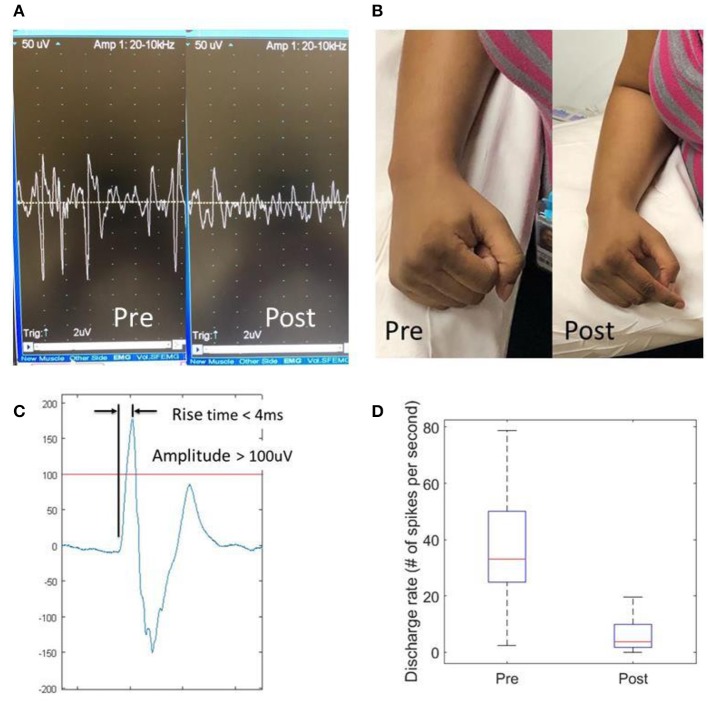
Effects of dry needling on finger flexor spasticity after stroke. **(A)** Pre- and post-dry needling screen snapshots of resting spastic finger flexor EMG readings; **(B)** resting hand postures pre- and post-dry needling; **(C)** a representative motor unit action potential (MUAP). An MUAP must have a spike amplitude >100 uV with a rise time <4 ms; **(D)** Discharge rate of MUAPs pre- and post-dry needling.

Intramuscular needle EMG readings were exported to a PC for further processing using Matlab (MathWorks Inc., Natick, USA). A custom program was developed in order to identify and locate MUAP spikes. An MUAP spike was defined as any spontaneous discharge with a magnitude >100 microvolt and a rise time < 4 microsecond (Figure 1C) (8). Frequency of spontaneous MUAP spikes (i.e., the average number of spikes per second) over a 10-s period was calculated.

### Statistical Analysis

Paired *t*-test and Wilcoxon signed-rank test were performed to analyze the dry needling effects (PRE vs. POST) on interval measures (e.g., resting position) and ordinal measures (e.g., MAS score), respectively. A significance level of 0.05 was used.

## Results

Participants reported immediate relief of muscle tightness after dry needling (Figure 1B). The pain caused by dry needling was tolerated well by all participants. Immediately after needling, the PIP joints rested in a less flexed position (PRE vs. POST: 105.0 ± 5.8 vs. 112.1 ± 7.0 degrees, *p* = 0.036), while the resting positions for the DIP (113.6 vs. 125.3 degrees, *p* = 0.115) and MCP (120.5 vs. 120.3 degrees, *p* = 0.971) joints remained the same. The FDS muscle was felt less tight to palpation (3.4 ± 0.2 vs. 1.9 ± 0.2, *p* < 0.001) on a 1–5 taut band tightness scale. The MAS score decreased for FDS (2.3 ± 0.4 vs. 1.2 ± 0.4, *p* = 0.017) and FDP (2.2 ± 0.5 vs. 1.3 ± 0.5, *p* = 0.029), but no changes in MAS for the MCP joint (1.8 ± 0.5 vs. 1.3 ± 0.4, *p* = 0.17). There was no difference in PROM before and after dry needling. In majority of patients (7 out of 10), these joints could be passively ranged to the full extension, i.e., a ceiling effect. However, for those patients with residual voluntary finger extension (*n* = 2), active ROM increased by 23.5 degrees at the PIP joint, 10 degrees at the DIP joint and 10 degrees at the MCP joint. The needle EMG recordings showed that the spikes of spontaneous MUAPs decreased from 41.6 ± 5.5 spikes/s to 6.7 ± 2.2 spikes/s (*p* = 0.002), an 84% reduction after dry needling (Figures 1A,D). However, the pre-needling spike frequency was not correlated to MAS or resting position of the FDS muscles.

## Discussion

In our study, dry needling effectively and immediately reduced finger flexor spasticity, improved resting joint position and active range of motion in chronic stroke survivors with finger flexor spasticity. These effects were accompanied with a significant reduction of MUAP discharge rate of finger flexors by 84%.

Dry needling has been shown to break down muscle fibers and temporarily deplete acetylcholine neurotransmitters in animal studies (9), but EMG study in humans is rare. Our data show that dry needling is able to decrease MUAP spikes in humans. Spontaneous MUAP spikes are known to be reflective of motor units that are firing spontaneously or at subthreshold levels such that these motor units can easily be triggered by stretching or voluntary activation (2). Therefore, it is likely that concomitant reduction of finger flexor spasticity is resulted from temporary depletion of neurotransmitters. Though some muscle fibers were broken down by dry needling and such breakdown may contribute to MUAP reduction, the extent of MUAP reduction (84% reduction) and transit effects of spasticity reduction by dry needling (7) strongly argue against this relation.

On the other hand, it is known that trigger points develop as a result of excessive accumulation of acetylcholine at the motor endplate and intracellular Ca^++^ (10). Accumulated acetylcholine in the spastic muscles is likely to potentiate development of trigger points. Furthermore, muscle overactivity from sustained hyperexcitability and/or spontaneous firing of motor units could further facilitate and maintain trigger points in the spastic muscles. In turn, trigger points contribute to spastic hypertonia in a vicious cycle. The results of reduced muscle tightness and improved voluntary finger extension support the existence of trigger points in the spastic muscles and that breakdown of taut bands in trigger points contribute at least partly to the post-needling effects. The results also show that 30 s of dry needling has immediate effect on spasticity. Therefore, we believe that a combination of dry needling and botulinum toxin injection can enhance the outcome. The duration of the effect and the long-term cost/benefit of dry needling will be investigated in our future work by introducing a control group.

## Conclusion

Dry needling to the spastic finger flexors leads to immediate spasticity reduction, increased active range of motion, and decreased frequency of spontaneous motor unit firing spikes. The results suggest that (latent) trigger points may exist in spastic muscles and they contribute partly to spastic hypertonia of finger flexors in chronic stroke.

## Data Availability Statement

The datasets generated for this study are available on request to the corresponding author.

## Ethics Statement

The studies involving human participants were reviewed and approved by the IRB committee, UTHealth. The ethics committee waived the requirement of written informed consent for participation.

## Author Contributions

SL was responsible for conceptualization. AB and SL were responsible for experiments and data collection. AB, ZL, PZ, and SL were responsible for data analysis and interpretation. ZL, PZ, and SL were responsible for manuscript draft and critical revision. All authors approved the final version.

### Conflict of Interest

The authors declare that the research was conducted in the absence of any commercial or financial relationships that could be construed as a potential conflict of interest.
